# Monitored anaesthesia care without endotracheal intubation and general anaesthesia in pulsed field atrial fibrillation ablation

**DOI:** 10.1093/ehjopen/oeag070

**Published:** 2026-04-27

**Authors:** Weijia Wang, Swecha Goel, Susan J Estrada, Elliot Hwang, Jacob Obholz, Qiying Dai, Humberto J Vidaillet, Sanjay Kumar, Param P Sharma

**Affiliations:** Department of Cardiology, Marshfield Clinic Health System, 1000 N Oak Ave, Marshfield, WI 54449, USA; Department of Cardiology, Marshfield Clinic Health System, 1000 N Oak Ave, Marshfield, WI 54449, USA; Department of Anesthesia, Marshfield Clinic Health System, 1000 N Oak Ave, Marshfield, WI 54449, USA; Department of Cardiology, Marshfield Clinic Health System, 1000 N Oak Ave, Marshfield, WI 54449, USA; Department of Cardiology, Marshfield Clinic Health System, 1000 N Oak Ave, Marshfield, WI 54449, USA; Department of Cardiology, Mayo Clinic Health System, 700 West Ave S, La Crosse, WI 54601, USA; Department of Cardiology, Marshfield Clinic Health System, 1000 N Oak Ave, Marshfield, WI 54449, USA; Department of Cardiology, Marshfield Clinic Health System, Weston, WI 54476, USA; Department of Cardiology, Marshfield Clinic Health System, 1000 N Oak Ave, Marshfield, WI 54449, USA

**Keywords:** Atrial fibrillation, Pulsed field ablation, Anaesthesia

## Abstract

**Aims:**

Pulsed field ablation for atrial fibrillation offers shorter procedural times and improved safety compared with thermal ablation, but the optimal anaesthesia strategy remains uncertain. Most centres in the USA use general anaesthesia with endotracheal intubation, whereas monitored anaesthesia care without endotracheal intubation may be a safe and efficient alternative.

**Objectives:**

To compare procedural efficiency and safety between monitored anaesthesia care and general anaesthesia for pulsed field ablation.

**Methods and results:**

Consecutive adults who underwent pulsed field ablation for atrial fibrillation between 2024 and 2025 at Marshfield Clinic Health System were included. The anaesthesia approach was determined by operator preference. Procedural times, medication use, complications, and patient tolerance were recorded prospectively. Among 200 patients, 100 received monitored anaesthesia care without endotracheal intubation, and 100 received general anaesthesia. The mean age was 67 years, 42% were female, and the mean body mass index was 33 kilograms per square metre. Monitored anaesthesia care shortened case duration (77 vs. 95 min; *P* < 0.001) and laboratory time (118 vs. 153 min; *P* = 0.002) and required fewer adjunct medications (*P* < 0.001). Twenty-five procedures (25%) were challenging due to cough or motion but were all completed safely. Complications occurred in one monitored anaesthesia care case and six general anaesthesia cases (*P* = 0.054). Pulmonary vein isolation was achieved in all patients.

**Conclusion:**

Monitored anaesthesia care without endotracheal intubation was safe, efficient, and resource-sparing compared with general anaesthesia for pulsed field atrial fibrillation ablation.

## Graphical Abstract

**Graphical Abstract:**
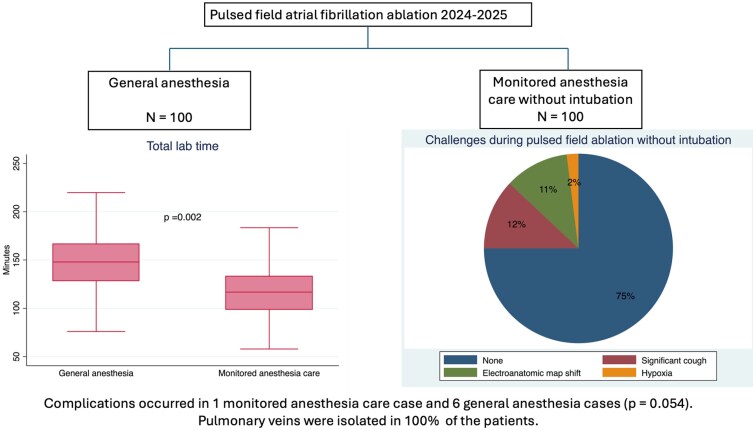

## Introduction

Pulmonary vein isolation for atrial fibrillation is the most commonly performed catheter ablation in the USA, with procedural volumes steadily increasing nationwide.^1^ Pulsed field ablation has been associated with a favourable safety profile and shorter procedural metrics than thermal ablation.^2,3^ Anaesthesia practice for pulsed field ablation varies: Most centres in the USA use general anaesthesia with endotracheal intubation as well as neuromuscular blockade, while deep sedation has been reported in non-US centres.^4,5^ General anaesthesia entails airway instrumentation and sometimes invasive arterial monitoring and can be associated with blood pressure lability. Ketamine-based deep sedation has been reported in European centres with encouraging outcomes.^6^ Compared to radiofrequency and cryoballoon ablations, pulsed field ablation can trigger skeletal muscle contractions and procedural discomfort. On the other hand, the ongoing anaesthesia workforce shortages complicate scheduling and access to care.^7^ Therefore, choosing an anaesthesia approach that balances safety, efficiency, and comfort is important.

In this report, we compared monitored anaesthesia care without endotracheal intubation and general anaesthesia with endotracheal intubation in consecutive patients undergoing pulsed field ablation for atrial fibrillation at a tertiary care centre in the USA.

## Methods

### Study sample

From August 2024 to September 2025, consecutive cases of pulsed field ablation performed in Marshfield Clinic Health System were examined.

Inclusion criteria were adults (≥18 years) who underwent pulsed field ablation for atrial fibrillation at Marshfield Clinic Health System. Three electrophysiologists (authors W.W., P.S., and S.K.) performed all procedures. Anaesthesia practice varied by operator: W.W. routinely scheduled atrial fibrillation ablations with monitored anaesthesia care without endotracheal intubation; P.S. scheduled ablations under general anaesthesia with endotracheal intubation; S.K. employed either approach based on clinical considerations such as body mass index and presence of sleep apnoea. All participants provided informed written consent for the ablation procedure. Study protocols were approved by the Institutional Review Boards at the Marshfield Clinic Health System.

All patients underwent atrial fibrillation ablation under anaesthesia administered by an anaesthesiology team. Anaesthetic strategy consisted of either general anaesthesia with endotracheal intubation or monitored anaesthesia care without intubation. The electrophysiology operator proposed the preferred anaesthesia strategy, but the final decision was made by the anaesthesiologist. To ensure transparency, we included all consecutive patients undergoing pulsed field ablation who were initially proposed by the electrophysiology operator for monitored anaesthesia care without endotracheal intubation.

Patients were identified retrospectively via the institutional clinical database. To ensure a representative sample, we utilized a consecutive enrolment strategy for both the monitored anaesthesia care and general anaesthesia groups. All patients undergoing atrial fibrillation ablation from August 2024 to September 2025 were evaluated. The first 100 consecutive patients in each group meeting inclusion criteria were included.

### Monitored anaesthesia care

During monitored anaesthesia care, continuous monitoring included noninvasive blood pressure, electrocardiography, pulse oximetry, respiratory rate, and capnography (end-tidal carbon dioxide). Supplemental oxygen was routinely administered. Capnography was used primarily for trend monitoring and early detection of hypoventilation rather than absolute end-tidal carbon dioxide quantification, recognizing its limitations during monitored anaesthesia care due to mouth breathing and oxygen dilution. No closed-loop or target-controlled anaesthesia delivery systems were used. Sedation depth was intended to achieve deep sedation while maintaining spontaneous ventilation, consistent with American Society of Anesthesiologists definitions.

Sedation was provided using titrated intravenous agents at the discretion of the anaesthesia team. Propofol was the primary sedative, administered as boluses or continuous infusion to achieve deep sedation while maintaining spontaneous ventilation. Adjunctive low-dose opioids and/or benzodiazepines were used selectively to enhance procedural tolerance. Dexmedetomidine was used at the discretion of the anaesthesiologist. Ketamine was rarely used. Roughly 10 min prior to ablation, the electrophysiologist notified the anaesthesia team to deepen sedation. Some anaesthesiologists added a lidocaine infusion at that time to optimize patient comfort. Lidocaine was started with a loading dose of 100 milligrams (approximating 1.5 milligrams per kilogram), followed by a continuous infusion of 2 milligrams per minute (approximating 1.5–2.0 milligrams per kilogram per hour until the ablation ended).

### Ablation procedures

All vascular accesses were obtained under ultrasound and fluoroscopic guidance. Transseptal puncture was performed with intracardiac echocardiography guidance. Intravenous heparin was administered to maintain an activated clotting time above 300–350 s. Three-dimensional electroanatomic mapping of the left atrium was performed using either the CARTO (Biosense Webster) or NavX (Abbott) system. A pulsed field ablation catheter (PulseSelect, Medtronic) was used to achieve wide antral isolation of all four pulmonary veins with 8–10 applications delivered per vein. Posterior wall and cavotricuspid isthmus ablation were performed in select cases. Entrance and exit block were confirmed in all targeted veins. At the conclusion of the procedure, haemostasis was obtained using either a vascular occlusion device or a figure-of-eight suture.

### Data abstraction

Demographic information and clinical data were abstracted from the electronic medical record. Procedural details were obtained from procedure notes by the electrophysiologists, anaesthesia reports, and procedure supporting document by the nursing staff. Procedural complications were monitored until hospital discharge.

Immediately following the procedure, the operating electrophysiologist documented each patient’s tolerance to monitored anaesthesia care without endotracheal intubation. When any uncertainty arose during data abstraction regarding intraprocedural events, clarification was obtained directly from the operator.

Case duration was defined as the time of needle stick for access to the time of sheath removal. Total lab time was defined as the time of patient entering the electrophysiology lab to the time of departure.

Challenging cases during monitored anaesthesia care were defined as episodes of significant coughing, displacement of the three-dimensional electroanatomic map that interfered with pulsed field ablation lesion annotation, any clinically relevant respiratory compromise (hypoxia and/or inadequate ventilation), or any other difficult situations.

### Statistical analysis

Baseline characteristics between the monitored anaesthesia care and general anaesthesia groups were compared using the Chi-squared test or Kruskal–Wallis test for categorical variables and the Student’s *t*-test or Wilcoxon rank-sum test for continuous variables. Case duration and laboratory time were assessed with analysis of variance for continuous variables. Associations between clinical factors and challenging cases (defined as significant cough, mapping shifts, or respiratory compromise) were evaluated using logistic regression.

To minimize the impact of inter-operator differences, a sensitivity analysis was performed including only cases conducted by the operator who contributed the largest number of cases.

To account for baseline differences between the monitored anaesthesia care and general anaesthesia groups, multivariable linear regression models were constructed for procedural duration and total lab time. These models adjusted for sex (as a categorical variable) and body mass index (as a continuous variable). Adjusted mean values were calculated using predictive margins and are reported as mean ± standard error.

All statistical analyses were performed with Stata/IC 14.2, and a two-sided *P*-value <0.05 was considered statistically significant.

The data underlying this article will be shared on reasonable request to the corresponding author.

## Results

### Study population

Between 2024 and 2025, 200 consecutive patients who underwent pulsed field ablation were included in the study (100 with general anaesthesia and 100 with monitored anaesthesia care). The two groups were similar in age and comorbidities. Female sex was more frequent among patients who received monitored anaesthesia care (49% vs. 35%, *P* = 0.045), and body mass index was higher in those treated with general anaesthesia (34.2 ± 7.8 vs. 31.5 ± 6.6 kg/m^2^, *P* = 0.008). Tobacco use was more common among patients receiving monitored anaesthesia care (13% vs. 1%, *P* < 0.001). Other baseline variables, including kidney function, CHA_2_DS_2_-VASc score, left ventricular ejection fraction, and major comorbidities, were comparable. Pulmonary vein isolation alone was more frequent with general anaesthesia (84% vs. 60%, *P* < 0.001), whereas posterior wall isolation and left atrial roof line was more common with monitored anaesthesia care (*Table 1*). There was no significant difference between the two groups in use of rate control agents, antiarrhythmics, or oral anticoagulation before the procedure (see Supplementary material online, *Table S1*).

**Table 1:** Baseline characteristics of patients undergoing pulsed field ablation by type of anaesthesia

Variable	General anaesthesia (*n* = 100)	Monitored anaesthesia care (*n* = 100)	*P*-value
Age, median (IQR), y	71.0 (64.0–77.0)	69.0 (63.0–76.0)	0.53
Female sex, *n* (%)	35 (35.0)	49 (49.0)	0.045
Body mass index, mean (SD), kg/m^2^	34.2 (7.8)	31.5 (6.6)	0.008
Atrial fibrillation type, *n* (%)			
Paroxysmal	54 (54.0)	42 (42.0)	0.089
Persistent	46 (46.0)	58 (58.0)	
eGFR, median (IQR), mL/min/1.73 m^2^	77.5 (61.0–88.5)	78.5 (64.0–95.0)	0.40
Obstructive sleep apnoea, *n* (%)	39 (39.0)	37 (37.0)	0.77
Chronic obstructive pulmonary disease, *n* (%)	15 (15.0)	9 (9.0)	0.19
Asthma, *n* (%)	5 (5.0)	5 (5.0)	1.00
Heart failure, *n* (%)	26 (26.0)	36 (36.0)	0.13
Coronary artery disease, *n* (%)	33 (33.0)	25 (25.0)	0.21
Hypertension, *n* (%)	76 (76.0)	65 (65.0)	0.088
Diabetes mellitus, *n* (%)	26 (26.0)	19 (19.0)	0.24
Alcohol use, *n* (%)	55 (55.0)	59 (59.0)	0.57
Tobacco use, *n* (%)	1 (1.0)	13 (13.0)	<0.001
CHA_2_DS_2_-VASc score, median (IQR)	3.0 (2.0–4.0)	3.0 (2.0–4.0)	0.73
Left ventricular ejection fraction, median (IQR), %	58.5 (54.0–64.0)	59.0 (52.5–63.0)	0.69
Left atrial volume index, median (IQR), mL/m^2^	35.0 (27.0–43.0)	34.0 (27.0–41.0)	0.47
Ablation type, *n* (%)			
Pulmonary vein isolation only	84 (84.0)	60 (60.0)	<0.001
Pulmonary vein isolation plus	16 (16.0)	40 (40.0)	
Redo ablation, *n* (%)	20 (20.0)	14 (14.0)	0.26
Concurrent CTI ablation, *n* (%)	33 (33.0)	25 (25.0)	0.21
Intraprocedural cardioversion, *n* (%)	38 (38.0)	48 (48.5)	0.14
ASA physical status, *n* (%)			0.084
II	3 (3.0)	11 (11.0)	
III	84 (84.0)	78 (78.0)	
IV	13 (13.0)	11 (11.0)	

eGFR, estimated glomerular filtration rate; CTI, cavotricuspid isthmus; ASA, American Society of Anesthesiologists; SD, standard deviation; IQR, interquartile range. Pulmonary vein isolation plus indicates pulmonary vein isolation plus posterior wall isolation or left atrial roof line. Continuous variables are reported as mean ± standard deviation or median (interquartile range) as appropriate. Categorical variables are presented as number (percentage).

### Procedural details

**Figure 1:**
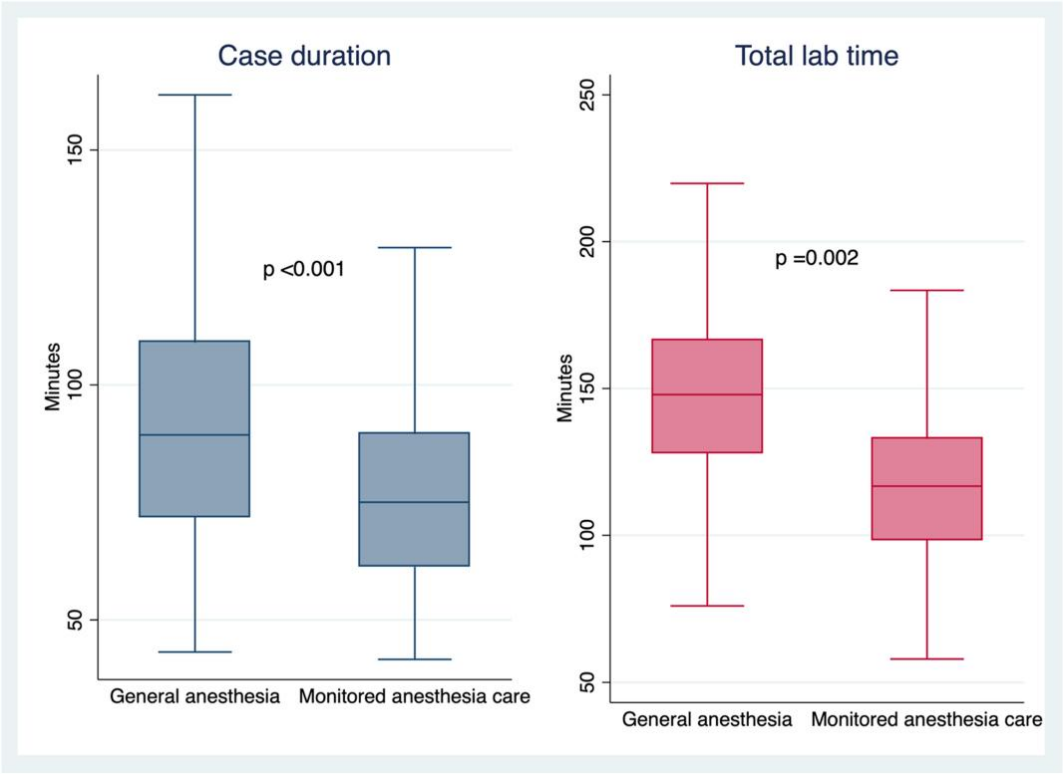
Case duration and total lab time by anaesthesia.

Pulmonary vein isolation was achieved in all 200 patients (100%). Procedural times were significantly longer with general anaesthesia vs. monitored anaesthesia care: case duration 95 ± 32 vs. 77 ± 21 min (*P* < 0.001) and total lab time 153 ± 35 vs. 118 ± 27 (*P* < 0.001) (*Figure 1*).

When we examined pulmonary vein isolation only cases (84 cases in the general anaesthesia group and 60 cases in the monitored anaesthesia care group), the findings were similar: case duration 95 ± 33 vs. 74 ± 21 min (*P* < 0.001) and total lab time 153 ± 35 vs. 116 ± 28 (*P* < 0.001). When limited to cases performed by a single operator, general anaesthesia was associated with longer case duration (86 ± 32 min vs. 75 ± 20 min; *P* = 0.018) and longer total laboratory time (147 ± 36 min vs. 116 ± 26 min; *P* < 0.001) compared with monitored anaesthesia care.

After adjusting for sex and body mass index, the monitored anaesthesia care group remained significantly more efficient. The adjusted mean case duration was 77 ± 3 min vs. 94 ± 3 min for general anaesthesia (*P* < 0.001), and total laboratory time was 118 ± 3 min vs. 153 ± 3 min (*P* < 0.001).

Fluoroscopy time differed between the monitored anaesthesia care and general anaesthesia groups, with mean values of 9 ± 4 min and 18 ± 11 min, respectively (*P* < 0.001). In a sensitivity analysis limited to cases performed by a single operator, fluoroscopy time was 8 ± 3 min in the monitored anaesthesia care group and 10 ± 5 min in the general anaesthesia group (*P* = 0.03).

One patient (1%) initially scheduled for monitored anaesthesia care was converted to general anaesthesia with endotracheal intubation prior to the procedure at the request of the anaesthesiologist due to his practice preference. Fourteen patients (14%) in the monitored anaesthesia care group underwent the procedure with a laryngeal mask airway for airway protection.

In the monitored anaesthesia care group, the median lowest respiratory rate was seven breaths per minute (interquartile range: 6–10). In the general anaesthesia group, where rates were controlled via mechanical ventilation, the median lowest rate was nine breaths per minute (interquartile range: 8–10).

**Figure 2:**
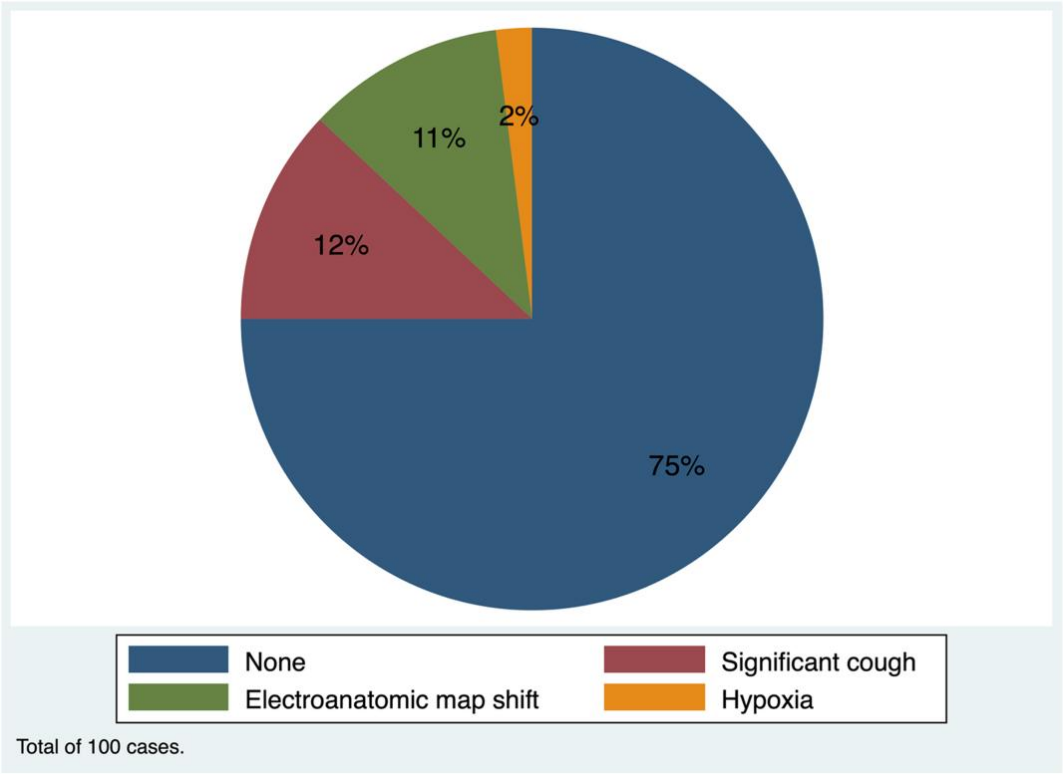
Challenges during pulsed field atrial fibrillation ablation under monitored anaesthesia care without endotracheal intubation.

In the monitored anaesthesia care group, 25 patients (25%) were classified as challenging by the operator (*Figure 2*). Among them, 16 patients (16%) had significant cough. Eleven patients (11%) exhibited substantial three-dimensional electroanatomic map instability, precluding accurate lesion annotation using the Carto system and necessitating reliance on fluoroscopic guidance. Two patients (2%) experienced respiratory compromise in the monitored anaesthesia care group: one with a transient respiratory pause and oxygen saturation <70% and another with excessive secretions and oxygen saturation <80%. Both cases were successfully managed with placement of a laryngeal mask airway. Transient oxygen desaturation to <85% occurred in 3 patients (3 of 100, 3%) in the monitored anaesthesia care group and in 0 patients (0 of 100, 0%) in the general anaesthesia group. All episodes were promptly recognized and managed without any clinical sequelae.

Use of anaesthetic and adjunct medications differed between the two anaesthesia strategies (*Table 2*). Intravenous lidocaine, fentanyl, rocuronium, ondansetron, and dexamethasone were administered less frequently in the monitored anaesthesia care group (*P* < 0.001 for all). In contrast, dexmedetomidine and ketamine were used more often in the monitored anaesthesia care group (*P* = 0.033 and *P* = 0.030, respectively). Propofol and midazolam were used in nearly all cases across both groups. There were also no statistically significant differences in the use of vasopressors, anticholinergics, or antiemetic agents such as ephedrine, glycopyrrolate, metoclopramide, norepinephrine, or phenylephrine.

**Table 2:** Intravenous medications used between general anaesthesia and monitored anaesthesia care

Medication	General anaesthesia (*n* = 100)	Monitored anaesthesia care (*n* = 100)	*P*-value
Lidocaine	93 (93.0%)	74 (74.0%)	<0.001
Propofol	98 (98.0%)	99 (99.0%)	0.56
Dexmedetomidine	37 (37.0%)	52 (52.0%)	0.033
Ketamine	1 (1.0%)	7 (7.0%)	0.030
Fentanyl	91 (91.0%)	52 (52.0%)	<0.001
Midazolam	16 (16.0%)	23 (23.0%)	0.21
Rocuronium	95 (95.0%)	0 (0.0%)	<0.001
Ondansetron	90 (90.0%)	30 (30.0%)	<0.001
Dexamethasone	60 (60.0%)	8 (8.0%)	<0.001
Ephedrine	14 (14.0%)	6 (6.0%)	0.059
Metoclopramide	0 (0.0%)	1 (1.0%)	0.32
Glycopyrrolate	16 (16.0%)	12 (12.0%)	0.41
Norepinephrine	2 (2.0%)	0 (0.0%)	0.16
Phenylephrine	88 (88.0%)	78 (78.0%)	0.060

No clinical variables or intraoperative drug use was associated with the challenging monitored anaesthesia cases (see Supplementary material online, *Tables S2* and *S3*).

A total of 7 complications (4% out of 200) were observed. One event (1% out of 100) occurred in the monitored anaesthesia care group, consisting of minor groin access bleeding. Six events (6% out of 100) occurred in the general anaesthesia group, including three cases of minor groin access bleeding, one transient pericarditis, one cardiac tamponade requiring pericardiocentesis, and one episode of fluid overload requiring intravenous diuresis. The complication rate trended lower in the monitored anaesthesia care group compared with the general anaesthesia group (1% vs. 6%, *P* = 0.054).

For the monitored anaesthesia care group, no patient reported recollection of pain or discomfort during the procedure. Post-procedural delirium was not observed in either the general anaesthesia or monitored anaesthesia care groups during postanaesthesia period.

## Discussion

In this study of 200 consecutive pulsed field ablation procedures for atrial fibrillation performed at a tertiary centre in the USA, monitored anaesthesia care without endotracheal intubation was safe and effective. Compared with general anaesthesia, monitored anaesthesia care without endotracheal intubation was associated with shorter procedure times and substantially reduced use of adjunct anaesthetic and supportive agents. Approximately one-quarter of procedures performed with monitored anaesthesia care were challenging due to coughing or respiratory motion, but all were managed successfully without major adverse events. These findings support the use of monitored anaesthesia care as a safe, efficient, and resource-sparing alternative to general anaesthesia for pulsed field ablation particularly in resource-limited settings.

Atrial fibrillation catheter ablation is rapidly increasing in demand. Pulsed field ablation requires less procedural time than traditional thermal energy sources and eliminates concerns about oesophageal injury and phrenic nerve paralysis.^2^ These advantages are accelerating procedural growth across both rural and urban settings, including hospital-based programmes and ambulatory surgical centres.^8^ The shortage of anaesthesiologists^9^ remains a bottleneck to expanding access to atrial fibrillation ablation. In this context, our study is timely, demonstrating that monitored anaesthesia care without endotracheal intubation can improve procedural efficiency, maintain excellent safety, and help broaden access to atrial fibrillation ablation.

General anaesthesia has traditionally been the preferred modality for atrial fibrillation ablation in the USA because of improved catheter stability which is particularly important for radiofrequency ablation.^10^ However, it requires endotracheal intubation, invasive blood pressure monitoring, and additional preparation time. Prior studies have described sedation strategies for pulsed field ablation, including the feasibility of electrophysiologist-led deep sedation in selected settings and real-world registry data characterizing anaesthesia and sedation practices.^11,12^ A recent meta-analysis of patients undergoing atrial fibrillation ablation found no difference in atrial arrhythmia recurrence between general anaesthesia and sedation.^13^ Deep sedation for pulsed field ablation has been increasingly reported in European studies using either the Farapulse (Boston Scientific, MA) or Affera (Medtronic) systems.^4,5,14^ In contrast, our study provided experience using the PulseSelect (Medtronic) system in a US population.

The advantages of monitored anaesthesia care without endotracheal intubation over general anaesthesia are clear. It shortened procedure and laboratory times by approximately 20% and reduced the use of adjunctive medications such as ondansetron, neuromuscular blocking agents, dexamethasone, and fentanyl. It also eliminated potential complications associated with arterial line placement and urinary catheterization, which are often required during general anaesthesia. Given its excellent safety profile, monitored anaesthesia care without endotracheal intubation can be considered a first-line anaesthesia approach for pulsed field ablation when performed by experienced operators.

Approximately one-quarter of procedures with monitored anaesthesia care were considered challenging by the operator. Sixteen per cent of patients with monitored anaesthesia care experienced significant coughing likely due to phrenic or diaphragmatic stimulation. Effective communication with the anaesthesia team is essential: first, to anticipate that mild coughing or movement may occur and, second, to consider transient deepening of sedation prior to ablation when needed. In about 10% of cases with monitored anaesthesia care, electroanatomic maps on the Carto system shifted during energy delivery, and repositioning of surface patches or magnets did not resolve the issue. We routinely mark the pulmonary vein ostia fluoroscopically during pre-ablation mapping and rely on fluoroscopy to confirm catheter–tissue contact regardless of whether the map shifts. Desaturation occurred in 2% of patients with monitored anaesthesia care related to respiratory pause or airway secretions. Transient bronchospasm has also been described with pulsed field ablation in the prior report.^15^ Although no variables were predictive of challenging cases, concerns for hypoxaemia remain. In patients with severe chronic obstructive pulmonary disease, asthma, or markedly elevated body mass index, coordination with anaesthesiology is advised to assess the need for general anaesthesia and enhanced airway control. Most complications were vascular access related and may not be attributable to anaesthesia modality.

### Study strength and limitations

The main strength of this study was that the cohort included a broad spectrum of body mass index and comorbidities, enhancing its real-world relevance and reflecting nonurban practice settings where anaesthesiology resources are often limited.

However, several limitations warrant discussion. Anaesthesia modality was not randomized and was influenced by operator preference. Because operator experience, training background, and anaesthesia approach were closely linked, observed differences in procedural efficiency may partially reflect operator-level factors rather than anaesthesia modality alone. Also, most monitored anaesthesia care cases were performed by a single operator (WW, 93 of 100), so the shorter procedural duration observed may partly reflect operator-specific factors. Nevertheless, all operators followed comparable workflows, including transseptal puncture, pre-ablation mapping, pulmonary vein isolation, and post-ablation confirmation. In addition, adjunct posterior wall or roof–line ablation prolongs procedure duration, and the imbalance between groups confounds the interpretation of efficiency differences. The classification of ‘challenging’ cases was subjective. No validated instrument was used to assess patient comfort, although no patient reported pain or distress. An equivalent metric was not available for patients undergoing general anaesthesia, as general anaesthesia is expected to minimize coughing and patient movement owing to deeper sedation and neuromuscular blockade. This ‘challenging’ case designation was descriptive, intended to capture real-world intraprocedural difficulties during monitored anaesthesia care rather than serve as a comparative endpoint. The procedural success was defined as complete pulmonary vein isolation. This study was designed to evaluate procedural feasibility and safety. Further studies are needed to assess long term arrhythmia outcome. Also, the complications were not reviewed by an independent adjudication committee. Complications were reviewed by three authors (S.G., E.H., and J.O.) and verified by one (WW). These findings apply to the PulseSelect system and may not be directly generalizable to other pulsed field platforms. Because cumulative anaesthetic dosing was not consistently documented in a standardized manner, we were unable to report total drug exposure.

Finally, while this study demonstrates the feasibility, it was not powered to provide a definitive comparative assessment of rare but major complications associated with atrial fibrillation ablation. The effects of different anaesthesia strategies for pulsed field atrial fibrillation ablation on procedural safety, patient-reported outcomes, and atrial fibrillation recurrence require evaluation in larger and randomized studies with clinical follow-up.

## Conclusions

Monitored anaesthesia care without endotracheal intubation was feasible and efficient for pulsed field atrial fibrillation ablation. Although some cases required escalation to a laryngeal mask airway or were challenging due to cough and movement, this non-intubated strategy appears safe in appropriately selected patients.

## Clinical perspectives

### Clinical competencies

Monitored anaesthesia care without endotracheal intubation allows safe and effective pulsed field atrial fibrillation ablation while reducing procedure time and resource use. This approach may enhance laboratory efficiency, address anaesthesia workforce constraints, and broaden access to pulsed field ablation.

### Translational outlook

Randomized trials are needed to validate these findings and guide optimal anaesthesia selection for pulsed field atrial fibrillation ablation.

## Supplementary Material

oeag070_Supplementary_Data
