# Supplementary material for: Monitored anaesthesia care without endotracheal intubation and general anaesthesia in pulsed field atrial fibrillation ablation
Source: Eur Heart J Open. 2026 Apr 27;6(2):oeag070. doi: 10.1093/ehjopen/oeag070 (PMC13159995; doi:10.1093/ehjopen/oeag070)
Supplement: oeag070_Supplementary_Data [file oeag070_supplementary_data.docx]

**Supplementary material**

**Table S1. Baseline Medication Use by Type of Anesthesia**

| **Medication Class** |  | **General Anesthesia**  **(n = 100)** | **Monitored Anesthesia Care**  **(n = 100)** | **p Value** |
| --- | --- | --- | --- | --- |
| **Beta-blocker use, n (%)** |  |  |  | 0.48 |
| None |  | 20 (20.0) | 21 (21.0) |  |
| Atenolol |  | 2 (2.0) | 0 (0.0) |  |
| Bisoprolol |  | 1 (1.0) | 0 (0.0) |  |
| Carvedilol |  | 3 (3.0) | 5 (5.0) |  |
| Metoprolol |  | 74 (74.0) | 73 (73.0) |  |
| **Calcium-channel blocker use, n (%)** |  |  |  | 0.60 |
| None |  | 89 (89.0) | 88 (88.0) |  |
| Verapamil |  | 0 (0.0) | 1 (1.0) |  |
| Diltiazem |  | 11 (11.0) | 11 (11.0) |  |
| **Digoxin use, n (%)** |  |  |  | 0.70 |
| No |  | 97 (97.0) | 96 (96.0) |  |
| Yes |  | 3 (3.0) | 4 (4.0) |  |
| **Antiarrhythmic drug use, n (%)** |  |  |  | 0.37 |
| None |  | 69 (69.0) | 72 (72.0) |  |
| Amiodarone |  | 12 (12.0) | 12 (12.0) |  |
| Dofetilide |  | 10 (10.0) | 5 (5.0) |  |
| Dronedarone |  | 6 (6.0) | 6 (6.0) |  |
| Flecainide |  | 1 (1.0) | 4 (4.0) |  |
| Propafenone |  | 0 (0.0) | 1 (1.0) |  |
| Sotalol |  | 2 (2.0) | 0 (0.0) |  |
| **Oral anticoagulant use, n (%)** |  |  |  | 0.69 |
| None (patient choice) |  | 1 (1.0) | 0 (0.0) |  |
| Apixaban |  | 72 (72.0) | 76 (76.0) |  |
| Dabigatran |  | 1 (1.0) | 0 (0.0) |  |
| Rivaroxaban |  | 13 (13.0) | 11 (11.0) |  |
| Warfarin |  | 13 (13.0) | 13 (13.0) |  |

**Note:** Baseline medication use among patients undergoing pulsed field ablation for atrial fibrillation, stratified by anesthesia type. Categorical variables are presented as number (percentage).

### Table S2. Association Between Clinical Variables and Challenging Monitored Anesthesia Care Cases

| **Variable** | **Odds Ratio** | **95% Confidence Interval** | **p-value** |
| --- | --- | --- | --- |
| Age (per tertile increase) | 1.1 | 0.7–2.0 | 0.627 |
| Female sex | 0.8 | 0.3–1.9 | 0.564 |
| BMI ≥ 35 kg/m² | 1.2 | 0.4–3.4 | 0.689 |
| Obstructive sleep apnea | 1.2 | 0.5–3.0 | 0.720 |
| Chronic obstructive pulmonary disease | 2.7 | 0.7–10.8 | 0.170 |
| Heart failure | 0.6 | 0.2–1.7 | 0.338 |
| Alcohol use | 1.7 | 0.6–4.3 | 0.293 |
| Tobacco use | 0.9 | 0.2–3.5 | 0.864 |
| Left ventricular ejection fraction (per tertile increase) | 1.3 | 0.7–2.3 | 0.380 |
| ASA physical status | 1.3 | 0.5–3.4 | 0.623 |

**Note:** **Challenging** monitored anesthesia care **cases were** defined by significant cough, electroanatomic map shifting, or respiratory compromise. Age and ASA physical status were analyzed both as continuous variables and as categorical variables (age by tertiles). ASA = American Society of Anesthesiologists.

### **Table S3. Association Between Intra-Operative Drug Use and Challenging** Monitored Anesthesia Care **Cases**

| **Drug** | **Odds Ratio** | **95% Confidence Interval** | **p-value** |
| --- | --- | --- | --- |
| Lidocaine | 0.9 | (0.3 – 2.4) | 0.792 |
| Propofol | 1.0 | (1.0 – 1.0) | — |
| Dexmedetomidine | 0.7 | (0.3 – 1.6) | 0.357 |
| Ketamine | 2.4 | (0.5 – 11.7) | 0.270 |
| Fentanyl | 1.2 | (0.5 – 3.1) | 0.644 |
| Midazolam | 0.6 | (0.2 – 1.8) | 0.341 |
| Phenylephrine | 1.7 | (0.5 – 5.5) | 0.406 |
| Rocuronium | 1.0 | (1.0 – 1.0) | — |
| Ondansetron | 2.3 | (0.9 – 6.0) | 0.082 |
| Dexamethasone | 1.0 | (0.2 – 5.3) | 1.000 |
| Ephedrine | 1.0 | (1.0 – 1.0) | — |
| Metoclopramide | 1.0 | (1.0 – 1.0) | — |
| Glycopyrrolate | 0.6 | (0.1 – 2.8) | 0.482 |
| Norepinephrine | 1.0 | (1.0 – 1.0) | — |

**Note: Challenging** monitored anesthesia care **cases were** defined by significant cough, electroanatomic map shifting, or respiratory compromise. Universally used drugs such as propofol or rocuronium yielded odds ratio of 1.0 without confidence intervals or p-values.
